# Pregnancy-Related Factors and Breast Cancer Risk for Women Across a Range of Familial Risk

**DOI:** 10.1001/jamanetworkopen.2024.27441

**Published:** 2024-08-26

**Authors:** Jasmine A. McDonald, Yuyan Liao, Julia A. Knight, Esther M. John, Allison W. Kurian, Mary Daly, Saundra S. Buys, Yun Huang, Caren J. Frost, Irene L. Andrulis, Sarah V. Colonna, Michael L. Friedlander, John L. Hopper, Wendy K. Chung, Jeanine M. Genkinger, Robert J. MacInnis, Mary Beth Terry

**Affiliations:** 1 Columbia University Irving Medical Center, New York, New York; 2 Lunenfeld-Tanenbaum Research Institute, Sinai Health, Toronto, Canada; 3 Stanford University School of Medicine, Stanford, California; 4 Fox Chase Cancer Center, Philadelphia, Pennsylvania; 5 University of Utah Health Sciences Center, Salt Lake City; 6 Ministry of Education, Shanghai Key Laboratory of Children’s Environmental Health, Xinhua Hospital, Shanghai Jiao Tong University School of Medicine and School of Public Health, Shanghai Jiao Tong University School of Medicine, Shanghai, China; 7 College of Social Work, The University of Utah, Salt Lake City; 8 Dalla Lana School of Public Health, University of Toronto, Toronto, Ontario, Canada; 9 University of Utah Health Huntsman Cancer Institute, Salt Lake City; 10 University of New South Wales, Sydney, New South Wales, Australia; 11 Melbourne School of Population and Global Health, The University of Melbourne, Parkville, Victoria, Australia; 12 Cancer Council Victoria, East Melbourne, Victoria, Australia; 13 Department of Molecular Genetics, University of Toronto, Toronto, Ontario, Canada

## Abstract

**Importance:**

Few studies have investigated whether the associations between pregnancy-related factors and breast cancer (BC) risk differ by underlying BC susceptibility. Evidence regarding variation in BC risk is critical to understanding BC causes and for developing effective risk-based screening guidelines.

**Objective:**

To examine the association between pregnancy-related factors and BC risk, including modification by a of BC where scores are based on age and BC family history.

**Design, Setting, and Participants:**

This cohort study included participants from the prospective Family Study Cohort (ProF-SC), which includes the 6 sites of the Breast Cancer Family Registry (US, Canada, and Australia) and the Kathleen Cuningham Foundation Consortium (Australia). Analyses were performed in a cohort of women enrolled from 1992 to 2011 without any personal history of BC who were followed up through 2017 with a median (range) follow-up of 10 (1-23) years. Data were analyzed from March 1992 to March 2017.

**Exposures:**

Parity, number of full-term pregnancies (FTP), age at first FTP, years since last FTP, and breastfeeding.

**Main Outcomes and Measures:**

BC diagnoses were obtained through self-report or report by a first-degree relative and confirmed through pathology and data linkages. Cox proportional hazards regression models estimated hazard ratios (HR) and 95% CIs for each exposure, examining modification by PARS of BC. Differences were assessed by estrogen receptor (ER) subtype.

**Results:**

The study included 17 274 women (mean [SD] age, 46.7 [15.1] years; 791 African American or Black participants [4.6%], 1399 Hispanic or Latinx participants [8.2%], and 13 790 White participants [80.7%]) with 943 prospectively ascertained BC cases. Compared with nulliparous women, BC risk was higher after a recent pregnancy for those women with higher PARS (last FTP 0-5 years HR for interaction, 1.53; 95% CI, 1.13-2.07; *P* for interaction < .001). Associations between other exposures were limited to ER-negative disease. ER-negative BC was positively associated with increasing PARS and increasing years since last FTP (*P* for interaction < .001) with higher risk for recent pregnancy vs nulliparous women (last FTP 0-5 years HR for interaction, 1.54; 95% CI, 1.03-2.31). ER-negative BC was positively associated with increasing PARS and being aged 20 years or older vs less than 20 years at first FTP (*P* for interaction = .002) and inversely associated with multiparity vs nulliparity (*P* for interaction = .01).

**Conclusions and Relevance:**

In this cohort study of women with no prior BC diagnoses, associations between pregnancy-related factors and BC risk were modified by PARS, with greater associations observed for ER-negative BC.

## Key Points

**Question:**

Does estimated absolute breast cancer (BC) risk modify the association between pregnancy-related factors and BC risk?

**Findings:**

In this cohort study of 17 274 women with no prior breast cancer diagnoses, higher breast cancer risk after a recent pregnancy was observed compared with nulliparous women, and risk was further increased with higher estimated absolute risk. These observed associations were greater for an estrogen receptor–negative diagnosis.

**Meaning:**

These findings suggest increased breast cancer risk from a recent pregnancy may be even greater for women at increasing levels of absolute breast cancer risk.

## Teaser

This cohort study evaluates the association between pregnancy-related factors and breast cancer risk, including modification by estimated absolute risk score.

## Introduction

Breast cancer (BC) incidence in women younger than 50 years has been increasing since 1976, with some estimates as high as a 2% annual change^1,2^ and notable differences in trends by BC molecular subtype.^3^ Changes in reproductive trends have been cited as a partial explanation for these incidence trends, as a later age at first birth, lower parity, and an earlier age at first menses are all associated with an increased risk of hormone receptor–positive tumors,^4,5,6^ while a later age at first birth is associated with a reduced risk of estrogen receptor (ER)–negative and triple-negative BC.^5,7^ Importantly, although parity decreases ER-positive BC risk in the long-term, in the short-term there is a risk from parity.^5,7^ Recent childbirth has been associated with a peak BC risk around 5 years. However, compared with nulliparous women, the increased BC risk may last more than 20 years for ER-positive BC, with no observable crossover inverse association for ER-negative BC.^8,9,10,11^ Breastfeeding duration is consistently associated with lower risk of both ER-positive and ER-negative BC, with studies supporting a greater risk reduction for ER-negative BC.^4,12^ Studies in women who carry pathogenic or likely pathogenic variants (PVs) in BC susceptibility genes (eg, *BRCA1* and *BRCA2*) suggest that underlying BC risk modifies these associations.^13,14^ Less is known, however, about the role of the absolute risk spectrum, including women at higher risk related to their family history but without an identified *BRCA1 *or *BRCA2* PV. Understanding pregnancy-related risk factors across the absolute risk spectrum, including if women are at higher BC risk immediately following pregnancy, is critical to developing effective risk-based screening guidelines. Using the Prospective Family Study Cohort (ProF-SC), we examined the associations between pregnancy-related factors and BC risk, focusing on modification by predicted absolute risk scores (PARS).

## Methods

### Study Sample

ProF-SC^15^ includes 6 international sites of the Breast Cancer Family Registry (BCFR) in the US, Canada, and Australia^16^ and the Kathleen Cuningham Foundation Consortium for research into Familial Breast Cancer (KCF).^17,18^ In brief, cohort members were followed up prospectively for cancer diagnoses with systematic follow-ups 10 years and 15 years after enrollment in the BCFR. Follow-up occurred every 3 years for KCF participants. We obtained each participant’s BC diagnosis through self-report or report by a first-degree relative.^15^ We confirmed 81% of incident invasive BC diagnoses through pathology reports or linkage with registries (cancer registry, national death index, and so forth). The BCFR and KCF conducted *BRCA1* and *BRCA2* germline analysis as previously described.^16,19,20^ The parent BCFR protocol and KCF were approved by their site-specific local ethics review board. All participants provided written informed consent before enrollment. For this study, we selected women who were enrolled before June 30, 2011, and were unaffected by BC at baseline (18 856 participants) and used follow-up data through March 2017.^21^ We excluded women lost to follow-up (196 participants), those whose diagnoses dates were missing (21 participants), and those who had a bilateral mastectomy before baseline (113 participants) or follow-up time less than 1 year (691 participants). We further excluded those whose age at baseline was 80 years or older (468 participants) and breast and ovarian analysis of disease incidence and carrier estimation algorithm (BOADICEA) BC 1-year risk was unknown (93 participants). For the analyses, we used epidemiology data downloaded with outcomes assessed through March 2017.

### Epidemiology Data

The BCFR and KCF used the same baseline questionnaires at study enrollment. The questionnaires assessed self-reported educational attainment, race, ethnicity, age and outcome of each pregnancy, breastfeeding history, and diagnosis of breast or ovarian cancer and breast or ovarian surgical procedures. Probands (ie, first person enrolled in each family) also completed a family history questionnaire that assessed histories of BC or other cancers in first- and second-degree relatives.

### Reproductive Data

We operationalized reproductive variables and created composite FTP-breastfeeding variables as previously described (see Table 1).^13^ The referent category for each analysis is indicated within the tables and figures. When appropriate, we also examined a linear term for the reproductive data, but as the associations did not change the direction, we only report the output for the categorical terms.

**Table 1.** Demographic and Pregnancy-Related Characteristics of Women in the Prospective Cohort Affected and Unaffected With Breast Cancer (BC)

Characteristic	Participants, No. (%)	
	Affected with BC (n = 943)	Unaffected with BC (n = 16 331)
Age at baseline interview, mean (SD), y	48.6 (12.2)	46.6 (15.2)
Age at censoring, mean (SD), y	55.9 (12.3)	57.1 (14.9)
Follow-up time, median (IQR), y	7.0 (4.0-10.0)	11.0 (6.0-14.0)
Year of birth		
<1940	202 (21.4)	3304 (20.2)
1940-1949	239 (25.3)	3000 (18.4)
1950-1959	261 (27.7)	3811 (23.3)
≥1960	241 (25.6)	6216 (38.1)
Race and ethnicity^a^		
African American/Black	29 (3.1)	762 (4.7)
Asian	33 (3.5)	607 (3.8)
Hispanic/Latinx (any race)	45 (4.8)	1354 (8.4)
White	807 (86.6)	12983 (80.4)
Other race and ethnicity^b^	18 (1.9)	448 (2.8)
Age at menarche, y^a^		
≤11	148 (15.8)	2764 (17.1)
12	243 (26.0)	3853 (23.9)
13	276 (29.5)	4530 (28.1)
≥14	269 (28.7)	4985 (30.9)
No. of FTP		
1	109 (11.6)	1874 (11.5)
2	319 (33.8)	4850 (29.7)
3	205 (21.7)	3416 (20.9)
≥4	140 (14.8)	2724 (16.7)
Nulliparous	170 (18.0)	3467 (21.2)
Age at first FTP among parous, y		
<20	107 (13.8)	2184 (17.0)
20-24	307 (39.7)	4907 (38.1)
25-29	203 (26.3)	3629 (28.2)
≥30	156 (20.2)	2144 (16.7)
Age, mean (SD)	24.9 (5.2)	24.5 (5.2)
Years since last FTP		
0-5	47 (5.0)	602 (3.7)
6-20	213 (22.6)	3278 (20.1)
≥21	513 (54.4)	8984 (55.0)
Nulliparous	170 (18.0)	3467 (21.2)
Breastfeeding duration among live births, mo		
0	148 (19.2)	2648 (20.6)
1-5	280 (36.3)	4354 (33.9)
6-12	158 (20.5)	2710 (21.1)
13-24	125 (16.2)	2033 (15.8)
>24	61 (7.9)	1110 (8.6)
Breastfeeding duration at last FTP, mo		
0	254 (26.9)	4285 (26.2)
1-5	270 (28.6)	4320 (26.5)
≥6	249 (26.4)	4259 (26.1)
Nulliparous	170 (18.0)	3467 (21.2)
*BRCA1* and *BRCA2* pathogenic and likely pathogenic variant status		
*BRCA1* positive, *BRCA2* negative	27 (2.9)	141 (0.9)
*BRCA1* positive, *BRCA2* not tested	76 (8.1)	411 (2.5)
*BRCA1* negative, *BRCA2* positive	19 (2.0)	80 (0.5)
*BRCA1* not tested, *BRCA2* positive	49 (5.2)	367 (2.2)
*BRCA1* negative, *BRCA2* negative	121 (12.8)	1326 (8.1)
*BRCA1* negative, *BRCA2* not tested	32 (3.4)	984 (6.0)
*BRCA1* not tested, *BRCA2* negative	125 (13.3)	2115 (13.0)
*BRCA1* not tested, *BRCA2* not tested	494 (52.4)	10 907 (66.8)
BOADICEA 1-y breast cancer risk (%), mean (SD)^c^	0.8 (0.9)	0.4 (0.6)

Abbreviations: BOADICEA, breast and ovarian analysis of disease incidence and carrier estimation algorithm; FTP, full-term pregnancy.

^a^ Missing data includes the following: race, 1.1% missing; age at menarche, 1.2% missing.

^b^ Other includes American Indian or Alaska Native, Pacific Islander, unspecified other race, and multirace.

^c^ Estimated absolute risk score was calculated by BOADICEA version 3, which uses multigenerational data on breast and ovarian cancer in relatives and genetic variants in breast cancer susceptibility genes (*BRCA1* and *BRCA2*).

### Predicted Absolute Risk Score

We used BOADICEA version 3, which uses multigenerational data on breast and ovarian cancer in relatives and PVs to estimate PARS for 1-year risk based on age^22,23^; details are published elsewhere.^24^ We did not use the more recent version of BOADICEA, which incorporates additional risk factors, including reproductive factors, as our purpose was to have an independent risk score to estimate interaction effects. The 1-year PARS was modeled continuously or based on median cut points or tertiles based on the unaffected population. To calculate a 5-year PARS, we multiplied the 1-year PARS by 5.

### Statistical Analysis

We conducted multivariable Cox proportional hazards regression modeling with age as a time scale to estimate hazard ratios (HR) and 95% CIs for 1-year PARS and each exposure variable. Person-time was calculated from the age at baseline questionnaire to the age at BC diagnosis, age at bilateral prophylactic mastectomy, age at last follow-up, age at death, or 80 years, whichever came first.^13,21,25^ We assessed multiplicative interaction by PARS and each exposure variable by including a cross-product term in the model and assessing the corresponding β coefficient using the Wald test. All models used a robust variance estimator to account for the correlation between family members. We stratified all models by birth cohort in 10-year categories (<1940, 1940-1949, 1950-1959, and ≥1960) and adjusted for baseline age (age at baseline questionnaire, continuous), study center, bilateral oophorectomy (yes or no) as a time-varying covariate, and age at menarche (≤11, 12, 13, or ≥14 years). We adjusted for race and ethnicity using a composite variable (Asian, Hispanic [any race], non-Hispanic Black, non-Hispanic White, or other race or ethnicity [includes American Indian or Alaksa Native, Pacific Islander, unspecified other race, and multirace]). Race was assessed in this study because non-Hispanic Black and Hispanic women are diagnosed at younger ages than non-Hispanic White women. Contingent on the pregnancy-related exposure of interest, we considered potentially confounding time-varying covariates such as number of FTP (0-1 or ≥2), number of live births (0-1 or ≥2), years since last FTP (nulliparous, 0-5, 6-20 or ≥21 years), and breastfeeding duration (0, 1-5, 6-12, 13-24, or >24 months). We conducted sensitivity analyses where we excluded women with a *BRCA1 *or *BRCA2* PV or with a prior diagnosis of any cancer before baseline, and we conducted analyses with confirmed invasive BC. We ran analyses by ER subtype, where the alternative BC subtype was censored at diagnosis. We generated quadratic splines to examine years since last FTP as a continuous nonlinear exposure, with knots placed at the 5th, 25th, 50th, 75th, and 95th percentiles.^8^ Within the quadratic spline model, we included a dichotomous indicator term where we set 0 for nulliparous women and an indicator variable for parous women. eTable in Supplement 1 summarizes the joint effect of years since last FTP × PARS and BC risk analyses. All statistical tests were 2-sided, and *P* values less than .05 were considered statistically significant. All analyses were performed with SAS software version 9.4 (SAS Institute). Data were analyzed from March 1992 to March 2017.

## Results

Among 17 274 women (mean [SD] age, 46.7 [15.1] years; 791 African American or Black participants [4.6%], 1399 Hispanic or Latinx participants [8.2%], and 13 790 White participants [80.7%]) with a median (range) follow-up of 10 (1-23) years, we observed 943 incident cases of BC over 177 754 person-years of follow-up (mean [SD] age at diagnosis of 55.9 [12.3] years; range, 26-80 years); 103 BC cases (11.0%) had a *BRCA1* PV and 68 (7.2%) had a *BRCA2* PV (Table 1).

The association between pregnancy-related factors and overall BC was modified by PARS (Table 2). Lower risk of BC was observed among multiparous women as PARS increased, but the finding was not statistically significant (*P *for interaction = .15); a significant decrease in BC risk was only observed for 4 or more FTPs vs nulliparous women (HR for interaction, 0.79; 95% CI, 0.65-0.97). The inverse association between PARS and multiparity was independent of breastfeeding at last FTP (Table 2). Individuals with a first FTP at age 30 years or older vs less than 20 years had an increased risk of BC (HR, 1.49; 95% CI, 1.10-2.02) and risk was elevated with a first FTP at age 25 or older with increasing PARS, but this finding was not statistically significant (*P* for interaction = .08). There was an association between increasing PARS (per single unit) in women who were within 5 years of last FTP and a positive BC risk (vs nulliparous women, HR for interaction, 1.53; 95% CI, 1.13-2.07) and an inverse BC risk in women for whom it had been 21 years since last FTP (vs nulliparous women, HR for interaction, 0.83; 95% CI, 0.71-0.97).

**Table 2.** Prospective Analyses for Pregnancy-Related Factors and 1-Year Breast Cancer Risk With Modification by Predicted Absolute Risk Score (PARS)

Pregnancy-related factor	No. overall/No. of events	HR (95% CI) without interaction	HR (95% CI) with interaction	*P* value for interaction
Parous^a^				
No	3521/165	1 [Reference]	1 [Reference]	NA
Parous	13 373/760	0.92 (0.77 to 1.09)	0.98 (0.81 to 1.19)	NA
PARS^b^	NA	1.50 (1.42 to 1.59)	1.62 (1.42 to 1.85)	NA
PARS × parous	NA	NA	0.91 (0.79 to 1.05)	.19
No. of FTP^a^				
Nulliparous	3521/165	1 [Reference]	1 [Reference]	NA
1 FTP	1939/106	1.00 (0.79 to 1.28)	1.05 (0.80 to 1.38)	NA
2 FTP	5084/316	0.98 (0.81 to 1.19)	1.01 (0.81 to 1.26)	NA
3 FTP	3547/202	0.87 (0.71 to 1.07)	0.89 (0.70 to 1.14)	NA
≥4 FTP	2803/136	0.73 (0.58 to 0.93)^c^	0.89 (0.67 to 1.18)	NA
PARS	NA	1.50 (1.42 to 1.59)	1.61 (1.41 to 1.84)	NA
PARS × 1 FTP	NA	NA	0.94 (0.75 to 1.17)	.15
PARS × 2 FTP	NA	NA	0.96 (0.82 to 1.12)	
PARS × 3 FTP	NA	NA	0.96 (0.82 to 1.13)	
PARS × ≥4 FTP	NA	NA	0.79 (0.65 to 0.97)^c^	
Age at first FTP (age as a categorical variable), y^d^				
<20	2253/105	1 [Reference]	1 [Reference]	NA
20-24	5112/302	1.24 (0.99 to 1.54)	1.27 (0.98 to 1.65)	NA
25-29	3755/199	1.18 (0.93 to 1.50)	1.07 (0.80 to 1.42)	NA
≥30	2253/154	1.67 (1.28 to 2.19)^c^	1.49 (1.10 to 2.02)^c^	NA
Nulliparous	3521/165	1.37 (0.99 to 1.90)	1.24 (0.86 to 1.77)	NA
PARS	NA	1.50 (1.42 to 1.59)	1.43 (1.24 to 1.65)	NA
PARS × 20-24	NA	NA	0.96 (0.81 to 1.13)	.08
PARS × 25-29	NA	NA	1.12 (0.94 to 1.33)	
PARS × ≥30	NA	NA	1.16 (0.96 to 1.40)	
PARS × nulliparous	NA	NA	1.13 (0.93 to 1.36)	
Age at first FTP (age as a continuous variable), y^d^				
Nulliparous vs parous	NA	1.11 (0.86 to 1.43)	1.04 (0.79 to 1.36)	NA
Age increase by 1 y	NA	1.03 (1.01 to 1.04)^c^	1.02 (1.00 to 1.03)	NA
PARS	NA	1.50 (1.42 to 1.59)	1.49 (1.40 to 1.58)	NA
PARS × nulliparous	NA	NA	1.08 (0.94 to 1.24)	.27
PARS × age	NA	NA	1.01 (1.00 to 1.02)^c^	.004
Time since last FTP (nulliparous reference), y^d^				
Nulliparous	3521/165	1 [Reference]	1 [Reference]	NA
0-5	631/47	1.33 (0.91 to 1.94)	1.11 (0.74 to 1.67)	NA
6-20	3411/209	1.01 (0.76 to 1.33)	0.93 (0.69 to 1.25)	NA
≥21	9331/504	0.94 (0.72 to 1.23)	1.12 (0.83 to 1.51)	NA
PARS	NA	1.50 (1.42 to 1.59)	1.61 (1.40 to 1.84)	NA
PARS × 0-5	NA	NA	1.53 (1.13 to 2.07)^c^	<.001
PARS × 6-20	NA	NA	1.14 (0.97 to 1.34)	
PARS × ≥21	NA	NA	0.83 (0.71 to 0.97)^c^	
Time since last FTP (age ≥21 reference), y^d^				
≥21	NA	1 [Reference]	1 [Reference]	NA
6-20	NA	1.07 (0.85 to 1.35)	0.83 (0.64 to 1.07)	NA
0-5	NA	1.41 (0.96 to 2.08)	0.99 (0.65 to 1.51)	NA
Nulliparous	NA	1.06 (0.81 to 1.39)	0.89 (0.66 to 1.20)	NA
PARS	NA	1.50 (1.42 to 1.59)	1.33 (1.23 to 1.45)	NA
PARS × 6-20	NA	NA	1.37 (1.21 to 1.56)^c^	<.001
PARS × 0-5	NA	NA	1.84 (1.38 to 2.46)^c^	
PARS × nulliparous	NA	NA	1.20 (1.03 to 1.40)^c^	
Breastfeeding duration, mo^g^				
No breastfeeding	2740/147	1 [Reference]	1 [Reference]	NA
1-5	4559/277	1.20 (0.98 to 1.47)	1.13 (0.89 to 1.43)	NA
6-12	2811/153	1.12 (0.89 to 1.41)	1.05 (0.81 to 1.38)	NA
13-24	2106/123	1.18 (0.92 to 1.52)	1.06 (0.80 to 1.42)	NA
>24	1148/59	1.07 (0.79 to 1.46)	1.03 (0.72 to 1.48)	NA
No live birth	3530/166	1.10 (0.84 to 1.44)	0.98 (0.73 to 1.32)	NA
PARS	NA	1.50 (1.42 to 1.59)	1.39 (1.22 to 1.58)	NA
PARS × 1-5	NA	NA	1.07 (0.91 to 1.27)	.64
PARS × 6-12	NA	NA	1.08 (0.89 to 1.31)	
PARS × 13-24	NA	NA	1.14 (0.96 to 1.35)	
PARS × >24	NA	NA	1.05 (0.85 to 1.29)	
PARS × no live birth	NA	NA	1.16 (0.97 to 1.39)	
No. of FTP and breastfeeding history^a^				
Nulliparous	3521/165	1 [Reference]	1 [Reference]	NA
1 FTP, never breastfeeding	583/32	0.90 (0.61 to 1.32)	1.04 (0.68 to 1.59)	NA
≥2 FTP, never breastfeeding	2164/116	0.80 (0.63 to 1.03)	0.90 (0.68 to 1.19)	NA
1 FTP, ever breastfeeding	1356/74	1.06 (0.81 to 1.40)	1.03 (0.76 to 1.41)	NA
≥2 FTP, ever breastfeeding	9270/538	0.93 (0.77 to 1.11)	0.98 (0.80 to 1.21)	NA
PARS	NA	1.50 (1.42 to 1.59)	1.62 (1.42 to 1.85)	NA
PARS × 1 FTP, never breastfeeding	NA	NA	0.82 (0.59 to 1.14)	.37
PARS × ≥2 FTP, never breastfeeding	NA	NA	0.87 (0.72 to 1.05)	
PARS × 1 FTP, ever breastfeeding	NA	NA	1.06 (0.83 to 1.36)	
PARS × ≥2 FTP, ever breastfeeding	NA	NA	0.92 (0.80 to 1.06)	
Breastfeeding duration at last FTP, mo^d^				
No breastfeeding	4446/250	1 [Reference]	1 [Reference]	NA
1-5	4515/267	1.13 (0.95 to 1.35)	1.03 (0.84 to 1.26)	NA
≥6	4412/243	1.07 (0.89 to 1.29)	0.93 (0.75 to 1.15)	NA
Nulliparous	3521/165	1.06 (0.81 to 1.38)	0.92 (0.69 to 1.23)	NA
PARS	NA	1.50 (1.42 to 1.59)	1.36 (1.23 to 1.49)	NA
PARS × 1-5	NA	NA	1.12 (0.98 to 1.29)	.049
PARS × ≥6	NA	NA	1.18 (1.04 to 1.35)^c^	
PARS × Nulliparous	NA	NA	1.19 (1.02 to 1.40)^c^	
No. of FTP and breastfeeding history at last FTP^a^				
Nulliparous	3521/165	1 [Reference]	1 [Reference]	NA
1 FTP, never breastfeeding at last FTP	584/32	0.90 (0.61 to 1.32)	1.04 (0.68 to 1.59)	NA
≥2 FTP, never breastfeeding at last FTP	3862/218	0.85 (0.69 to 1.05)	0.98 (0.77 to 1.24)	NA
1 FTP, ever breastfeeding at last FTP	1355/74	1.06 (0.81 to 1.40)	1.03 (0.76 to 1.41)	NA
≥2 FTP, ever breastfeeding at last FTP	7572/436	0.93 (0.77 to 1.11)	0.95 (0.77 to 1.18)	NA
PARS	NA	1.50 (1.42 to 1.59)	1.62 (1.42 to 1.85)	NA
PARS × 1 FTP, never breastfeeding at last FTP	NA	NA	0.82 (0.59 to 1.14)	.09
PARS × ≥2 FTP, never breastfeeding at last FTP	NA	NA	0.84 (0.72 to 0.99)^c^	
PARS × 1 FTP, ever breastfeeding at last FTP	NA	NA	1.06 (0.83 to 1.36)	
PARS × ≥2 FTP, ever breastfeeding at last FTP	NA	NA	0.96 (0.83 to 1.11)	

Abbreviations: FTP, full-term pregnancy; NA, not applicable; PARS, predicted absolute risk score.

^a^ Stratified on the birth cohort (birth year <1940, 1940-1949, 1950-1959, or ≥1960), adjusted for oophorectomy (yes or no), study site, race and ethnicity, and age at menarche.

^b^ PARS was estimated by the breast and ovarian analysis of disease incidence and carrier estimation algorithm version 3, which uses multigenerational data on breast and ovarian cancer in relatives and genetic variants in breast cancer susceptibility genes (*BRCA1* and *BRCA2*). PARS is a continuous variable in all shown models.

^c^ *P* values <.05.

^d^ Stratified on the birth cohort (birth year <1940, 1940-1949, 1950-1959, or ≥1960), adjusted for oophorectomy and number of full-term pregnancies (0-1 or ≥2), study site, race and ethnicity, and age at menarche.

^e^ Stratified on the birth cohort (birth year <1940, 1940-1949, 1950-1959, or ≥1960), adjusted for oophorectomy and number of live births (0-1 or ≥2), study site, race and ethnicity, and age at menarche.

**Figure 1.**
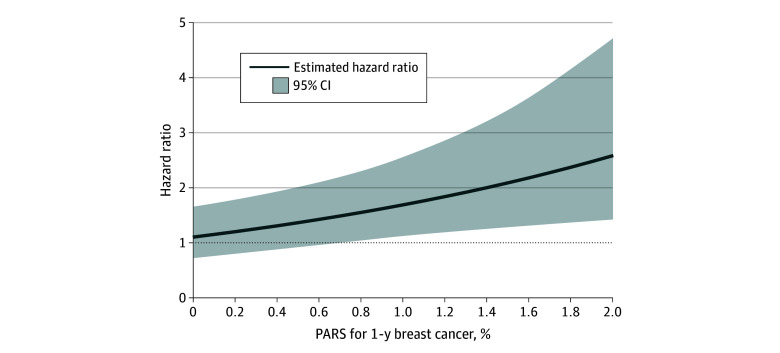
Breast Cancer Risk for 5 or Fewer Years Since Last Full-Term Pregnancy Compared With Nulliparous by 1-Year Predicted Absolute Risk Score (PARS) Results from a Cox proportional hazards regression of breast cancer risk with the following covariates: years since last full-term pregnancy, continuous 1-year breast and ovarian analysis of disease incidence and carrier estimation algorithm (BOADICEA) score, interaction between year since last full-term pregnancy and BOADICEA, stratified on the birth cohort, and further adjusted for number of full-term pregnancy, ever or never bilateral oophorectomy, race and ethnicity, age at menarche, and study site.

We observed that a 1-year PARS greater than 1% for women within 5 years of last FTP was associated with more than a 1.5-fold increased BC risk (Figure 1). Results were similar when restricted to confirmed invasive BC cases and when excluding those with a prior diagnosis of any cancer except nonmelanoma skin cancer (data not shown). Estimates were of greater magnitude but not statistically significant when removing *BRCA1 *or *BRCA2* PV carriers (PARS × ≤5 years since last FTP, HR for interaction, 1.98; 95% CI, 0.30-13.1).

**Figure 2.**
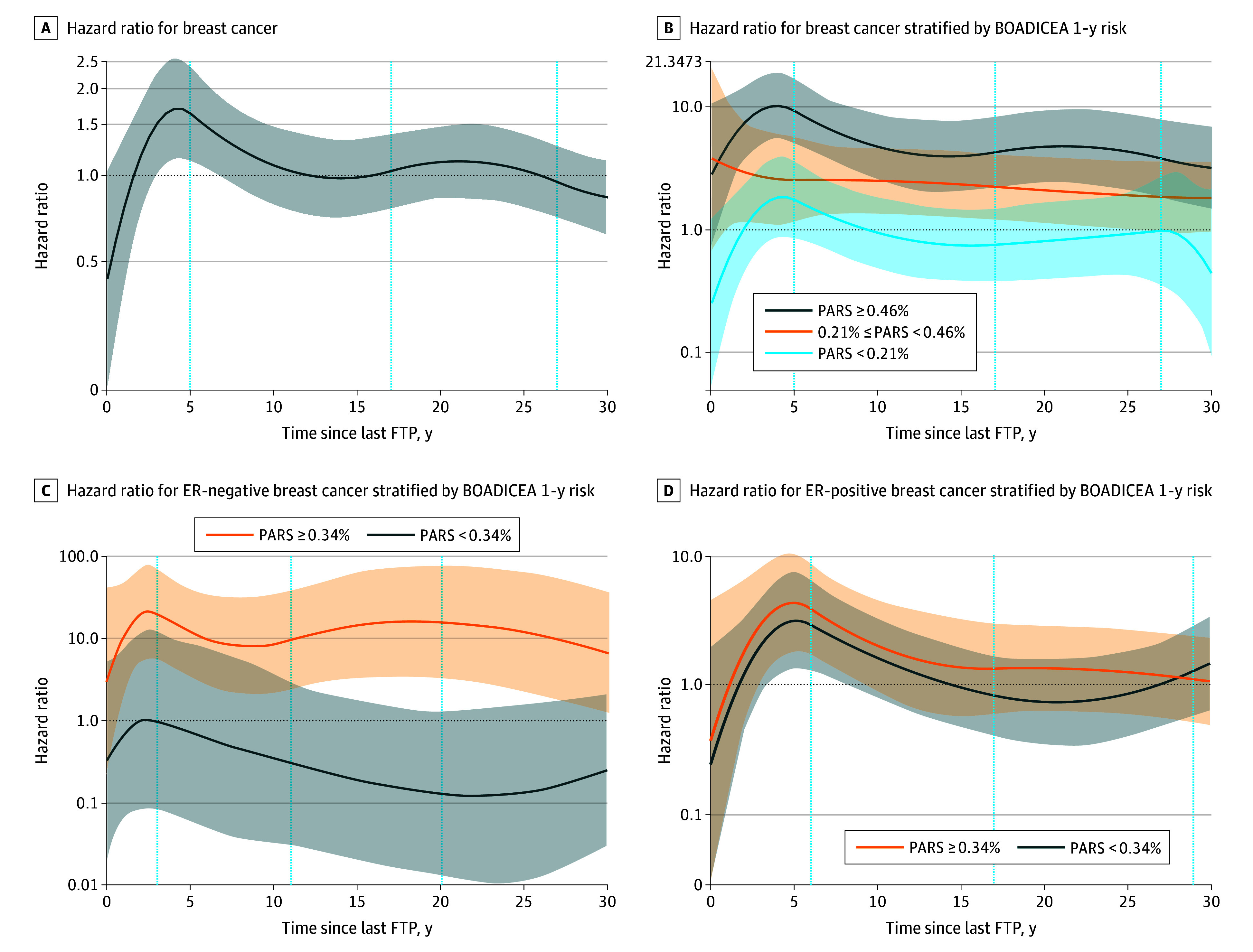
Breast Cancer Risk and Overall and Joint Effect of Years Since Last Full-Term Pregnancy (FTP) and Predicted Absolute Risk Score (PARS) Quadratic splines to examine year since last full-term pregnancy (FTP) as a continuous, nonlinear exposure, with knots placed at the 5th, 25th, 50th, 75th, and 95th percentiles of year since last FTP. Breast cancer risk (solid curved lines) and 95% CI (shaded areas). The spline model included nulliparous women where year since last FTP was set to 0 for nulliparous women and an indicator variable for parous women was included.

Figure 2A and B show the association between years since last FTP and BC risk overall and modeled by tertiles of 1-year PARS. Compared with nulliparous women, we observed a peak risk of BC within 5 years of the last FTP among women with a PARS of 0.21% or higher. Those with PARS of 0.46% or higher experienced a peak BC risk at 4 years since last FTP (HR for interaction, 10.2; 95% CI, 5.5-18.9); the lowest observed risk (HR for interaction, 3.20; 95% CI, 1.50-6.79), nearly 30 years after last FTP, remained significantly elevated, never crossing over to an inverse association during the observation period.

We observed no association between any breastfeeding variable and BC risk. PARS positively modified the associations between measures of breastfeeding duration at last FTP and 1-year BC risk, where breastfeeding measures were associated with slightly elevated BC risk (Table 2); however, associations did not remain after the removal of *BRCA1* or *BRCA2* PV carriers (breastfeeding ≥6 months at last FTP vs no breastfeeding at last FTP HR, 0.70; 95% CI, 0.38-1.30).

### Associations by ER Subtype Status

Observed associations were more pronounced in ER-negative BC (Table 3). Multiparity (≥4 vs nulliparous) was associated with a 2.3-fold increased risk of ER-negative BC (HR, 2.34; 95% CI, 1.09-5.01) but an inverse association with each unit increase with PARS (PARS × ≥4 vs nulliparous HR, 0.66; 95% CI, 0.48-0.90; *P *for interaction = .01), where the association was independent of breastfeeding at the last FTP. Age at first FTP was not associated with ER-negative BC (Table 3); however, compared with first FTP when aged less than 20 years, increasing PARS and advanced age at first FTP was associated with increased risk of ER-negative BC (*P *for interaction = .002). Similarly, years since last FTP was not associated with ER-negative BC; however, we observed an interaction with increasing PARS (Table 3). Compared with nulliparous women, increasing PARS for women with 5 or fewer years since last FTP was associated with a 54% increased risk of ER-negative BC (HR, 1.54; 95% CI, 1.03-2.31), and increasing PARS for women with 21 or more years since last FTP was associated with a 34% inverse risk of ER-negative BC (HR, 0.76; 95% CI, 0.58-0.99; *P *for interaction < .001). Figure 2C and D show the association between years since last FTP by ER-subtype, stratified by dichotomous 1-year PARS. Those with PARS of 0.34% or greater experienced a peak risk of ER-negative BC at 2.4 years since last FTP and no crossover to an HR less than 1 during the observation period.

**Table 3.** Estimated Associations by Estrogen Receptor (ER) Status for Pregnancy-Related Factors and 1-Year Breast Cancer Risk With Modification by Predicted Absolute Risk Score (PARS)

Factor	ER-negative			ER-positive		
	No. overall/No. of events	HR (95% CI) without interaction	HR (95% CI) with interaction	No. overall/No. of events	HR (95% CI) without interaction	HR (95% CI) with interaction
Parous^a^						
No	3521/19	1 [Reference]	1 [Reference]	3521/62	1 [Reference]	1 [Reference]
Parous	13 373/120	1.56 (0.94 to 2.57)	1.71 (0.98 to 2.97)	13373/298	0.92 (0.69 to 1.22)	0.94 (0.68 to 1.29)
PARS^b^	NA	1.90 (1.70 to 2.12)	2.07 (1.66 to 2.58)	NA	1.29 (1.17 to 1.43)	1.32 (1.06 to 1.65)
PARS × parous	NA	NA	0.91 (0.73 to 1.14)	NA	1.19 (0.89 to 1.59)	0.97 (0.76 to 1.24)
No. of FTP^a^						
Nulliparous	3521/19	1 [Reference]	1 [Reference]	3521/62	1 [Reference]	1 [Reference]
1	1939/10	0.95 (0.44 to 2.06)	0.80 (0.35 to 1.85)	1939/41	1.04 (0.70 to 1.55)	1.10 (0.71 to 1.73)
2	5084/57	1.83 (1.07 to 3.11)^c^	1.93 (1.05 to 3.56)^c^	5084/130	1.03 (0.76 to 1.41)	1.04 (0.73 to 1.47)
3	3547/33	1.66 (0.93 to 2.98)	1.76 (0.90 to 3.45)	3547/71	0.76 (0.53 to 1.07)	0.75 (0.50 to 1.14)
≥4	2803/20	1.34 (0.67 to 2.68)	2.34 (1.09 to 5.01)^c^	2803/56	0.76 (0.52 to 1.10)	0.75 (0.48 to 1.18)
PARS	NA	1.90 (1.70 to 2.12)	2.09 (1.67 to 2.61)	NA	1.29 (1.17 to 1.43)	1.31 (1.05 to 1.63)
PARS × 1	NA	NA	1.16 (0.84 to 1.59)^d^	NA	NA	0.90 (0.62 to 1.31)
PARS × 2	NA	NA	0.94 (0.74 to 1.20)^d^	NA	NA	0.99 (0.76 to 1.30)
PARS × 3	NA	NA	0.94 (0.72 to 1.24)^d^	NA	NA	1.00 (0.73 to 1.37)
PARS × ≥4	NA	NA	0.66 (0.48 to 0.90)^c^^,^^d^	NA	NA	1.00 (0.74 to 1.35)
Age at first FTP (age as a categorical variable), y^e^						
<20	2253/16	1 [Reference]	1 [Reference]	2253/44	1 [Reference]	1 [Reference]
20-24	5112/51	1.38 (0.77 to 2.48)	1.48 (0.75 to 2.91)	5112/113	1.07 (0.75 to 1.53)	1.09 (0.24 to 1.68)
25-29	3755/28	0.93 (0.48 to 1.79)	0.58 (0.27 to 1.25)	3755/79	1.12 (0.75 to 1.65)	1.09 (0.26 to 1.75)
≥30	2253/25	1.55 (0.74 to 3.22)	1.09 (0.48 to 2.49)	2253/62	1.67 (1.09 to 2.57)	1.52 (0.39 to 2.51)
Nulliparous	3521/19	1.35 (0.50 to 3.64)	1.00 (0.35 to 2.84)	3521/62	1.26 (0.74 to 2.17)	1.23 (0.37 to 2.23)
PARS	NA	1.89 (1.70 to 2.11)	1.70 (1.33 to 2.18)	NA	1.30 (1.17 to 1.44)	1.27 (0.16 to 1.62)
PARS × 20-24	NA	NA	0.92 (0.70 to 1.21)^f^	NA	NA	0.98 (0.15 to 1.32)
PARS × 25-29	NA	NA	1.38 (1.04 to 1.83)^c^^,^^f^	NA	NA	1.02 (0.17 to 1.41)
PARS × ≥30	NA	NA	1.29 (0.96 to 1.74)^f^	NA	NA	1.14 (0.20 to 1.60)
PARS × nulliparous	NA	NA	1.24 (0.91 to 1.68)^f^	NA	NA	1.03 (0.17 to 1.42)
Age at first FTP (age as a continuous variable), y^e^						
Nulliparous vs parous	NA	1.12 (0.49 to 2.55)	0.96 (0.42 to 2.21)	NA	1.10 (0.72 to 1.70)	1.09 (0.69 to 1.73)
Age increase by 1 y	NA	1.01 (0.97 to 1.06)	0.98 (0.93 to 1.03)	NA	1.03 (1.01 to 1.06)^c^	1.03 (1.00 to 1.06)
PARS	NA	1.89 (1.70 to 2.11)	1.86 (1.66 to 2.09)	NA	1.30 (1.17 to 1.44)	1.30 (1.16 to 1.45)
PARS × nulliparous	NA	NA	1.12 (0.89 to 1.40)	NA	NA	1.01 (0.79 to 1.29)
PARS × age	NA	NA	1.02 (1.01 to 1.04)^c^^,^^f^	NA	NA	1.01 (0.99 to 1.03)
Time since last FTP (nulliparous reference), y^e^						
Nulliparous	3521/19	1 [Reference]	1 [Reference]	3521/62	1 [Reference]	1 [Reference]
0-5	631/18	1.36 (0.55 to 3.35)	1.00 (0.39 to 2.54)	631/15	1.47 (0.74 to 2.92)	1.40 (0.68 to 2.85)
6-20	3411/43	0.94 (0.42 to 2.09)	0.80 (0.35 to 1.84)	3411/81	1.11 (0.69 to 1.78)	1.01 (0.61 to 1.67)
≥21	9331/59	0.74 (0.30 to 1.80)	1.21 (0.45 to 3.23)	9331/202	0.94 (0.62 to 1.44)	0.99 (0.62 to 1.58)
PARS	NA	1.89 (1.70 to 2.11)	2.05 (1.62 to 2.60)	NA	NA	1.28 (1.02 to 1.62)
PARS × 0-5	NA	NA	1.54 (1.03 to 2.31)^c^^,^^g^	NA	NA	1.15 (0.69 to 1.92)
PARS × 6-20	NA	NA	1.15 (0.89 to 1.50)^g^	NA	NA	1.17 (0.87 to 1.57)
PARS × ≥21	NA	NA	0.76 (0.58 to 0.99)^c^^,^^g^	NA	NA	0.95 (0.73 to 1.24)
Time since last FTP (age ≥21 reference), y^e^						
≥21	NA	1 [Reference]	1 [Reference]	NA	1 [Reference]	1 [Reference]
6-20	NA	1.27 (0.73 to 2.22)	0.66 (0.34 to 1.29)	NA	1.18 (0.82 to 1.70)	1.02 (0.69 to 1.53)
0-5	NA	1.84 (0.83 to 4.10)	0.83 (0.33 to 2.06)	NA	1.56 (0.79 to 3.09)	1.41 (0.68 to 2.91)
Nulliparous	NA	1.36 (0.56 to 3.32)	0.83 (0.31 to 2.20)	NA	1.06 (0.69 to 1.62)	1.01 (0.63 to 1.61)
PARS	NA	NA	1.55 (1.33 to 1.81)	NA	1.29 (1.17 to 1.43)	1.22 (1.06 to 1.41)
PARS × 6-20	NA	NA	1.53 (1.23 to 1.89)^c^^,^^g^	NA	NA	1.22 (0.97 to 1.54)
PARS × 0-5	NA	NA	2.04 (1.41 to 2.94)^c^^,^^g^	NA	NA	1.21 (0.74 to 1.95)
PARS × nulliparous	NA	NA	1.32 (1.01 to 1.73)^c^^,^^g^	NA	NA	1.05 (0.80 to 1.37)
Breastfeeding duration, mo^h^						
No breastfeeding	2740/16	1 [Reference]	1 [Reference]	2740/63	1 [Reference]	1 [Reference]
1-5	4559/41	1.34 (0.73 to 2.45)	1.46 (0.76 to 2.81)	4559/100	0.96 (0.70 to 1.33)	0.91 (0.62 to 1.33)
6-12	2811/27	1.30 (0.70 to 2.42)	1.11 (0.55 to 2.24)	2811/61	0.98 (0.69 to 1.41)	1.04 (0.68 to 1.60)
13-24	2106/24	1.36 (0.69 to 2.69)	1.09 (0.51 to 2.35)	2106/52	1.09 (0.73 to 1.63)	1.01 (0.64 to 1.60)
>24	1148/12	1.20 (0.54 to 2.70)	1.18 (0.47 to 2.93)	1148/22	0.91 (0.55 to 1.50)	1.01 (0.54 to 1.88)
No live birth	3530/19	1.18 (0.52 to 2.71)	1.02 (0.44 to 2.38)	3530/62	0.91 (0.58 to 1.40)	0.88 (0.54 to 1.44)
PARS	NA	1.90 (1.70 to 2.11)	1.81 (1.44 to 2.28)	NA	1.29 (1.17 to 1.44)	1.27 (1.02 to 1.57)
PARS × 1-5	NA	NA	0.92 (0.70 to 1.21)	NA	NA	1.07 (0.82 to 1.40)
PARS × 6-12	NA	NA	1.12 (0.84 to 1.48)	NA	NA	0.91 (0.63 to 1.32)
PARS × 13-24	NA	NA	1.18 (0.89 to 1.56)	NA	NA	1.11 (0.81 to 1.51)
PARS × >24	NA	NA	1.02 (0.75 to 1.39)	NA	NA	0.87 (0.49 to 1.55)
PARS × no live birth	NA	NA	1.15 (0.85 to 1.55)	NA	NA	1.04 (0.77 to 1.41)
No. of FTP and breastfeeding history^a^						
Nulliparous	3521/19	1 [Reference]	1 [Reference]	3521/62	1 [Reference]	1 [Reference]
1 FTP, never breastfeeding	583/2	0.68 (0.15 to 2.97)	0.39 (0.09 to 1.82)	583/12	0.89 (0.48 to 1.68)	1.04 (0.52 to 2.07)
≥2 FTP, never breastfeeding	2164/14	1.39 (0.68 to 2.84)	1.86 (0.84 to 4.11)	2164/51	0.95 (0.65 to 1.40)	0.95 (0.61 to 1.48)
1 FTP, ever breastfeeding	1356/8	1.07 (0.47 to 2.43)	0.95 (0.39 to 2.33)	1356/29	1.12 (0.72 to 1.75)	1.12 (0.67 to 1.87)
≥2 FTP, ever breastfeeding	9270/96	1.77 (1.05 to 2.99)^c^	2.01 (1.12 to 3.62)^c^	9270/206	0.88 (0.66 to 1.18)	0.89 (0.64 to 1.24)
PARS	NA	1.89 (1.70 to 2.11)	2.09 (1.67 to 2.61)	NA	1.29 (1.17 to 1.43)	1.32 (1.06 to 1.64)
PARS × 1 FTP, never breastfeeding	NA	NA	1.30 (0.91 to 1.88)	NA	NA	0.80 (0.49 to 1.31)
PARS × ≥2 FTP, never breastfeeding	NA	NA	0.78 (0.56 to 1.09)	NA	NA	1.00 (0.73 to 1.37)
PARS × 1 FTP, ever breastfeeding	NA	NA	1.14 (0.79 to 1.65)	NA	NA	1.01 (0.64 to 1.58)
PARS × ≥2 FTP, ever breastfeeding	NA	NA	0.89 (0.71 to 1.13)	NA	NA	0.98 (0.77 to 1.26)
Breastfeeding duration at last FTP, mo^b^						
No breastfeeding	4446/32	1 [Reference]	1 [Reference]	4446/99	1 [Reference]	1 [Reference]
1-5	4515/40	1.12 (0.70 to 1.78)	0.87 (0.52 to 1.47)	4515/105	1.09 (0.82 to 1.44)	1.01 (0.17 to 1.40)
≥6	4412/48	1.14 (0.72 to 1.81)	0.73 (0.43 to 1.25)	4412/94	1.01 (0.75 to 1.37)	1.01 (0.18 to 1.44)
Nulliparous	3521/19	1.15 (0.50 to 2.61)	0.79 (0.35 to 1.81)	3521/62	0.99 (0.64 to 1.53)	0.95 (0.23 to 1.53)
PARS	NA	1.90 (1.70 to 2.11)	1.55 (1.29 to 1.88)	NA	1.29 (1.17 to 1.44)	1.25 (0.10 to 1.47)
PARS × 1-5	NA	NA	1.20 (0.96 to 1.52)^d^	NA	NA	1.10 (0.13 to 1.40)
PARS × ≥6	NA	NA	1.39 (1.12 to 1.71)^c^^,^^d^	NA	NA	1.00 (0.14 to 1.31)
PARS × nulliparous	NA	NA	1.34 (1.03 to 1.76)^d^	NA	NA	1.06 (0.15 to 1.38)
No. of FTP and breastfeeding history at last FTP^a^						
Nulliparous	3521/19	1 [Reference]	1 [Reference]	3521/62	1 [Reference]	1 [Reference]
1 FTP, never breastfeeding at last FTP	584/2	0.89 (0.47 to 1.68)	0.40 (0.09 to 1.85)	584/12	0.89 (0.47 to 1.68)	1.03 (0.36 to 2.06)
≥2 FTP, never breastfeeding at last FTP	3862/30	0.88 (0.62 to 1.24)	2.52 (1.31 to 4.85)^c^	3862/87	0.88 (0.62 to 1.24)	0.90 (0.18 to 1.34)
1 FTP, ever breastfeeding at last FTP	1355/8	1.13 (0.72 to 1.75)	0.95 (0.39 to 2.33)	1355/29	1.13 (0.72 to 1.75)	1.12 (0.29 to 1.87)
≥2 FTP, ever breastfeeding at last FTP	7572/80	0.90 (0.67 to 1.21)	1.81 (0.99 to 3.31)	7572/170	0.90 (0.67 to 1.21)	0.90 (0.16 to 1.26)
PARS	NA	1.29 (1.17 to 1.44)	2.09 (1.67 to 2.61)	NA	1.29 (1.17 to 1.44)	1.32 (0.15 to 1.64)
PARS × 1 FTP, never breastfeeding at last FTP	NA	NA	1.29 (0.90 to 1.86)^f^	NA	NA	0.80 (0.20 to 1.31)
PARS × ≥2 FTP, never breastfeeding at last FTP	NA	NA	0.69 (0.52 to 0.90)^c^^,^^f^	NA	NA	0.96 (0.13 to 1.27)
PARS × 1 FTP, ever breastfeeding at last FTP	NA	NA	1.14 (0.79 to 1.65)^f^	NA	NA	1.01 (0.23 to 1.59)
PARS × ≥2 FTP, ever breastfeeding at last FTP	NA	NA	0.96 (0.76 to 1.21)^f^	NA	NA	1.00 (0.13 to 1.30)

Abbreviations: FTP, full-term pregnancy; HR, hazard ratio; NA, not applicable.

^a^ Stratified on the birth cohort (birth year <1940, 1940-1949, 1950-1959, or ≥1960), adjusted for oophorectomy (yes or no), study site, race, and age at menarche.

^b^ PARS was estimated by the breast and ovarian analysis of disease incidence and carrier estimation algorithm version 3, which uses multigenerational data on breast and ovarian cancer in relatives and genetic variants in breast cancer susceptibility genes. PARS is a continuous variable in all shown models. PARS is statistically significant across all models (*P* < .05).

^c^ *P* < .05.

^d^ Overall interaction term value: *P* < .05.

^e^ Stratified on the birth cohort (birth year <1940, 1940-1949, 1950-1959, or ≥1960), adjusted for oophorectomy and number of full-term pregnancies (0-1 or ≥2), study site, race, and age at menarche.

^f^ Overall interaction term value: *P* < .005.

^g^ Overall interaction term value: *P* < .001.

^h^ Stratified on the birth cohort (birth year <1940, 1940-1949, 1950-1959, or ≥1960), adjusted for oophorectomy and number of live birth (0-1 or ≥2), study site, race, and age at menarche.

Breastfeeding variables were overall null. However, women with increasing PARS who breastfed vs not breastfeeding at last FTP were at higher risk for ER-negative disease (*P *for interaction = .02) (Table 3).

## Discussion

With 943 incident BC cases identified among 17 274 women, this cohort study provides the first large-scale evaluation of pregnancy-related factors and BC risk across a spectrum of risk based on absolute estimated risk of BC that we know of. The associations between pregnancy-related factors and BC risk, with or without modification by PARS, were primarily limited to ER-negative BC. We observed a higher BC risk following a recent pregnancy based on a woman’s PARS, suggesting an important period for risk-based BC screening.

Time since last FTP and BC risk have been examined overall and among high-risk women defined by BC family history, *BRCA1* or *BRCA2* PV status, and polygenic risk score (PRS).^8,13,26^ In a pooled analysis of 15 prospective studies (with 18 826 incident BC diagnoses before age 55 years), authors observed a peak 1.8-fold increased risk of BC 4.6 years since most recent childbirth when compared with nulliparous women.^8^ In this same study, parous women with a BC family history vs nulliparous women without a BC family history had a 3.5-fold increased risk at 4.9 years since most recent childbirth. In addition, our prior pooled cohort study of 7970 *BRCA1* and 5135 *BRCA2* PV carriers found that the recency of last FTP was associated with increased BC risk among women with a *BRCA2* PV (≤5 years since last FTP vs nulliparous HR, 1.37; 95% CI, 1.06-1.78).^13^ In the current study, with increasing absolute BC risk as defined by a continuous PARS, recent pregnancy was associated with increased BC risk. Women with a 1-year risk of 0.21% or higher had a significant increased risk of BC within 5 years of last FTP with no observed crossover to an inverse association for at least 15 years from last FTP. However, women with a PARS of 0.46% or higher experienced no long-term inverse association from last FTP; at 30 years from last FTP, women with a PARS of 0.46% or higher retained a more than 3-fold increased risk of BC with greater associations for ER-negative disease. This observation parallels previous reports where, compared with nulliparous women, 2.2 years since most recent childbirth was associated with an approximately 2-fold increased risk of ER-negative BC where risk remained elevated for more than 30 years.^8^ When considering potential mechanisms, a prior study^26^ found a positive association between a 313-variant PRS and deficiency in breast involution as proxied by a higher amount of terminal duct lobular units; suggesting that the modification between PARS × recent pregnancy and BC risk may be mediated by a greater deficiency in breast involution.

Similar to past studies, we observed that the association with parity varied by BC subtype, with high parity being associated with a higher risk of ER-negative BC.^13,27,28,29^ The Black Women’s Health Study observed that increasing parity among women younger than 45 years was associated with an approximately 2-fold increased risk of ER-negative/PR-negative BC (≥3 births vs nulliparous).^30^ Using a case-control design within the BCFR, we also found an approximately 2-fold increased risk of ER-negative/PR-negative BC among multiparous premenopausal women (≥3 births vs nulliparous).^27^ In the current study we also found that high parity (≥4 FTP vs nulliparous) was associated with an approximately 2-fold increased risk of ER-negative BC; however, this association was mitigated by increasing PARS. This suggests that as absolute BC risk increases, multiparity may mitigate the risk of developing ER-negative BC.

We did not observe an overall association between age at first FTP and BC risk. In contrast, several studies have reported a positive association between older age at FTP and risk of ER-positive BC.^4,31,32,33,34^ A meta-analysis found that advanced age at first birth was associated with increased risk of luminal BC, but not with risk of triple-negative BC.^4^ In contrast, we observed a significant association between older ages at first FTP and increasing PARS with an increased risk of ER-negative BC. This suggests that with increasing absolute BC risk, women at a more advanced age at their first FTP carry a greater risk for ER-negative disease.

Models are used across clinical specialties to assess BC risk and inform surveillance strategies. The defined cutoffs for high-risk categories vary. A 5-year BC risk of 1.67% or greater is sometimes used to recommend chemoprevention.^35^ Our study observed that women with a 1-year PARS of 0.21% or higher are at peak BC risk within 5 years of last FTP, with risk lasting 15 years or longer. Based on current cutoffs, the women we observed with a 5-year PARS of 1.05% or greater would not be considered at high or moderate risk. Machine learning algorithms have also been shown to reclassify a woman’s BC risk as high or moderate, with the greatest reclassification impact on women younger than 50 years being recategorized to higher risk categories in comparison with current clinical models.^36^ The clinical utility of any model is dependent on sensitivity and specificity, where misclassification of high risk can have consequences. Given that women who have recently given birth are often undergoing closer medical surveillance for postnatal care, there is an opportunity to inform, develop, and evaluate surveillance strategies and messaging for women with a higher short-term risk of BC.

### Limitations

Limitations of this study include insufficient statistical power to stratify further by racial and ethnic groups^37,38^ and that the BOADICEA considers an underlying latent construct of polygenic risk, but we did not have polygenic risk available for the full cohort to examine this in this study.^39^ However, our findings support that the associations of pregnancy-related risk factors with BC are modified by absolute BC risk. Although not fully understood, the risks associated with ER-negative BC may be related to different hormonal pathways and/or nonhormonal factors in comparison with ER-positive BC. With increasing PARS, the recency of last FTP and a longer breastfeeding duration at last FTP were independently associated with increased risk of ER-negative BC. The latter finding could be spurious given breastfeeding is overall associated with a 4.3% risk reduction for every 12 months of breastfeeding, with greater benefit for ER-negative BC.^40^ However, the association between breastfeeding and BC risk varies by time and absolute risk. Nichols and colleagues^8^ similarly observed that cumulative breastfeeding history did not modify the association between years since most recent childbirth and overall BC risk. Moreover, longer duration of breastfeeding is protective among *BRCA1* PV carriers, but not *BRCA2* PV carriers.^13,41^ Nonetheless, our findings emphasize the importance of postpartum biology.^42,43,44,45^ Biologically, the postpartum window involves the involution of terminal ductal lobular units of the breast and a structural remodeling^46^; processes likened to wound healing, which are hypothesized to create a carcinogenic promoting or permissive mammary microenvironment (eg, expansion of the lymphatic vasculature, inflammation, and immune suppression).^46,47,48,49^ While preclinical evidence suggests that breastfeeding modifies the postpartum mammary microenvironment,^43,50^ epidemiological research on normal postlactational breast biology is lacking.

## Conclusions

This study suggests that pregnancy-related factors and BC risk are modified by estimated absolute risk of BC, with associations observed for ER-negative disease. Women with a recent pregnancy and greater underlying BC risk were at an elevated risk of developing ER-negative disease. Future studies that assess the clinical implications for this at-risk group (ie, screening modality and frequency) are needed.

## Notes

Supplement 1. **eTable. **Summary of PARS and Years Since Last FTP and Breast Cancer Risk

Supplement 2.
Data Sharing Statement

Supplement 3.
Nonauthor Collaborators
