# Supplementary material for: Pregnancy-Related Factors and Breast Cancer Risk for Women Across a Range of Familial Risk
Source: JAMA Netw Open. 2024 Aug 26;7(8):e2427441. doi: 10.1001/jamanetworkopen.2024.27441 (PMC13082433; doi:10.1001/jamanetworkopen.2024.27441)
Supplement: Supplement 1. — eTable. Summary of PARS and Years Since Last FTP and Breast Cancer Risk [file jamanetwopen-e2427441-s001.pdf]

Supplemental Online Content

McDonald JA, Liao Y, Knight JA, et al. Pregnancy-related factors and breast cancer risk for women across a range of familial risk. *JAMA Netw Open*. 2024;7(8):e2427441. doi:10.1001/jamanetworkopen.2024.27441

eTable. Summary of PARS and Years Since Last FTP and Breast Cancer Risk

This supplemental material has been provided by the authors to give readers additional information about their work.

© 2024 McDonald JA et al. *JAMA Network Open*.

eTable. Summary of PARS and Years Since Last FTP and Breast Cancer Risk

	Overall	ER-negative	ER-positive
**PARS*Years since last full-term pregnancy**			
PARS*<5	**1.53 (1.13 to 2.07)**	**1.54 (1.03 to 2.31)**	1.15 (0.69 to 1.92)
PARS*6-≤20	1.14 (0.97 to 1.34)	1.15 (0.89 to 1.50)	1.17 (0.87 to 1.57)
PARS*>20	**0.83 (0.71 to 0.97)**	**0.76 (0.58 to 0.99)**	0.95 (0.73 to 1.24)
**Quadratic spline stratified by PARS tertile/median (Peak risk of years since last full-term pregnancy)**			
PARS<0.21%	1.85 (0.88 to 3.88)	NA	NA
PARS 0.21%-<0.46%	3.82 (0.68 to 21.3)	NA	NA
PARS≥0.46%	**10.2 (5.50 to 18.9)**	NA	NA
PARS<0.34%	NA	1.04 (0.09 to 12.6)	3.15 (1.34 to 7.45)
PARS≥0.34%	NA	**21.2 (5.78 to 77.4)**	4.34 (1.82 to 10.3)

© 2024 McDonald JA et al. *JAMA Network Open*.
