# Supplementary material for: Pregnancy-Related Factors and Breast Cancer Risk for Women Across a Range of Familial Risk
Source: JAMA Netw Open. 2024 Aug 26;7(8):e2427441. doi: 10.1001/jamanetworkopen.2024.27441 (PMC13082433; doi:10.1001/jamanetworkopen.2024.27441)
Supplement: Supplement 2. — Data Sharing Statement [file jamanetwopen-e2427441-s002.pdf]

Data Sharing Statement

McDonald. Pregnancy-Related Factors and Breast Cancer Risk for Women Across a Range of Familial Risk. *JAMA Netw Open*. Published August 21, 2024.
doi:10.1001/jamanetworkopen.2024.27441

Data
Data available: Yes
Data types: Deidentified participant data
How to access data: https://www.bcfamilyregistry.org/data-sharing
When available: With publication

Supporting Documents
Document types: None

Additional Information
Who can access the data: https://www.bcfamilyregistry.org/data-sharing
Types of analyses: https://www.bcfamilyregistry.org/data-sharing
Mechanisms of data availability: https://www.bcfamilyregistry.org/data-sharing
Any additional restrictions: https://www.bcfamilyregistry.org/data-sharing
