# Supplementary material for: Pregnancy-Related Factors and Breast Cancer Risk for Women Across a Range of Familial Risk
Source: JAMA Netw Open. 2024 Aug 26;7(8):e2427441. doi: 10.1001/jamanetworkopen.2024.27441 (PMC13082433; doi:10.1001/jamanetworkopen.2024.27441)
Supplement: Supplement 3. — Nonauthor Collaborators [file jamanetwopen-e2427441-s003.pdf]

Supplemental Online Content: Nonauthor Collaborators
*First name, last name, and suffix (if applicable) are required and will appear in PubMed.

***Group Name(s):**							
***First Name and Middle Initial(s)**	***Last Name**	***Suffix (eg, Jr, III)**	Academic Degrees	Institution	Location (city, state/province, country)	Role or Contribution, eg, chair, principal investigator	Group (if more than 1 Group listed in the byline) and/or Subgroup (eg, Steering Committee)
David	Amor		MBBS, PhD				
Lesley	Andrews		MBBS				
Yoland	Antill		MBBS, PhD				
Rosemary	Balleine		MBBS, PhD FRCPA				
Jonathan	Beesley		PhD				
Ian	Bennett		MBBS, FRACS, FACS				
Michael	Bogwitz						
Simon	Bodek		PhD				
Leon	Botes						
Meagan	Brennan		MBBS,				
Melissa	Brown		PhD				
Michael	Buckley		FRACGP, FASBP, PhD				
Jo	Burke		PhD				
Phyllis	Butow		MBBS, PhD FRCPA				
Liz	Caldon		PhD				
Ian	Campbell		PhD				
Michelle	Cao		PhD				
Anannya	Chakrabarti		PhD				
Deepa	Chauhan						
Manisha	Chauhan						

Supplemental Online Content: Nonauthor Collaborators
*First name, last name, and suffix (if applicable) are required and will appear in PubMed.

***First Name and Middle Initial(s)**	***Last Name**	***Suffix (eg, Jr, III)**	Academic Degrees	Institution	Location (city, state/province, country)	Role or Contribution, eg, chair, principal investigator	Group (if more than 1 Group listed in the byline) and/or Subgroup (eg, Steering Committee)
Georgia	Chenevix-Trench		PhD				
Alice	Christian		GDip Gen Couns				
Paul	Cohen						
Alison	Colley		PhD				
Ashley	Crook		BSc(Hons) FHGSA				
James	Cui		MD, FRANZCOG,				
Eliza	Courtney		Dip Obs, BMBCh				
Margaret	Cummings		PhD				
Sarah-Jane	Dawson		GDip Gen Couns				
Anna	deFazio						
Martin	Delatycki		PhD				
Rebecca	Dickson						
Joanne	Dixon		MBBS, PhD FRCPA				
Stacey	Edwards		MBBS, PhD				
Gelareh	Farshid		PhD				
Andrew	Fellows		MBBS, PhD				
Georgina	Fenton		GDip Gen Couns				
Michael	Field						
James	Flanagan		MB ChB, FRACP				

Supplemental Online Content: Nonauthor Collaborators
*First name, last name, and suffix (if applicable) are required and will appear in PubMed.

***First Name and Middle Initial(s)**	***Last Name**	***Suffix (eg, Jr, III)**	Academic Degrees	Institution	Location (city, state/province, country)	Role or Contribution, eg, chair, principal investigator	Group (if more than 1 Group listed in the byline) and/or Subgroup (eg, Steering Committee)
Peter	Fong		PhD				
Laura	Forrest		MBBS, PhD FRCPA				
Stephen	Fox		PhD				
Juliet	French		GDip Gen Couns				
Michael	Friedlander						
Clara	Gaff		MBBS, PhD				
Mike	Gattas		PhD				
Peter	George		MBBS, FRACP				
Sian	Greening		PhD				
Marion	Harris		BSc, MBChB, FRCPath,				
Stewart	Hart		FFSc, FRCPA, DPhil				
Philip	Harraka		PhD				
Nick	Hayward		MBChB, MRCP				
John	Hopper		FRACP PhD				
Cass	Hoskins						
Clare	Hunt		PhD				
Paul	James		MBBS, FRACP				
Mark	Jenkins		PhD				
Alexa	Kidd		GDip Gen Couns				

Supplemental Online Content: Nonauthor Collaborators
*First name, last name, and suffix (if applicable) are required and will appear in PubMed.

***First Name and Middle Initial(s)**	***Last Name**	***Suffix (eg, Jr, III)**	Academic Degrees	Institution	Location (city, state/province, country)	Role or Contribution, eg, chair, principal investigator	Group (if more than 1 Group listed in the byline) and/or Subgroup (eg, Steering Committee)
Judy	Kirk						
Jessica	Koehler		MBBS, FRACP				
James	Kollias		MBBS, FRACS				
Sunil	Lakhani		PhD				
Mitchell	Lawrence		PhD				
Jason	Lee		PhD				
Shuai	Li		BSci				
Geoff	Lindeman		GDip Gen Couns				
Jocelyn	Lippey						
Lara	Lipton		MBBS, PhD				
Liz	Lobb		PhD				
Sherene	Loi		MBBS, MRCP,				
Graham	Mann		MRCGP				
Deborah	Marsh		MBBS, PhD,				
Sue Anne	McLachlan		GDip Gen Couns				
Bettina	Meiser						
Roger	Milne		MBBS, FRACS				
Sophie	Nightingale						
Shona	O'Connell		PhD				
Sarah	O'Sullivan						
David	Gallego Ortega						
Nick	Pachter		BSc, MBBS, Ph D				

Supplemental Online Content: Nonauthor Collaborators
*First name, last name, and suffix (if applicable) are required and will appear in PubMed.

***First Name and Middle Initial(s)**	***Last Name**	***Suffix (eg, Jr, III)**	Academic Degrees	Institution	Location (city, state/province, country)	Role or Contribution, eg, chair, principal investigator	Group (if more than 1 Group listed in the byline) and/or Subgroup (eg, Steering Committee)
Jia-Min	Pang		FRACP FA HMS FAA				
Gargi	Pathak		MBBS, Ph D				
Briony	Patterson						
Amy	Pearn		BSc, MBBS, Ph D				
Kelly	Phillips						
Ellen	Pieper		PhD				
Susan	Ramus						
Edwina	Rickard		MBBS, PhD, FRACP				
Abi	Ragunathan		PhD				
Bridget	Robinson		MBBS, PhD				
Mona	Saleh		PhD				
Anita	Skandarajah		PhD				
Elizabeth	Salisbury		MB ChB, MS, FRACS				
Christobel	Saunders		GDip Gen Couns				
Jodi	Saunus						
Peter	Savas		GDip Gen Couns				
Rodney	Scott						
Clare	Scott		PhD				
Adrienne	Sexton		MB ChB				
Joanne	Shaw						
Andrew	Shelling						

Supplemental Online Content: Nonauthor Collaborators
*First name, last name, and suffix (if applicable) are required and will appear in PubMed.

***First Name and Middle Initial(s)**	***Last Name**	***Suffix (eg, Jr, III)**	Academic Degrees	Institution	Location (city, state/province, country)	Role or Contribution, eg, chair, principal investigator	Group (if more than 1 Group listed in the byline) and/or Subgroup (eg, Steering Committee)
Shweta	Srinivasa		GDip Gen Couns				
Peter	Simpson						
Melissa	Southey		GDip Gen Couns				
Amanda	Spurdle						
Jessica	Taylor		MBBS, MD, FRACP				
Renea	Taylor		GDip Gen Couns				
Heather	Thorne						
Alison	Trainer						
Kathy	Tucker		GDip Gen Couns				
Jane	Visvader						
Logan	Walker						
Rachael	Williams		BMedSc MD FRACP				
Ingrid	Winship		PhD				
Mary Ann	Young						
Milita	Zaheed		MBBS, FRCPA				
			MB BS, FRCS, FRACS,				
			FAAHMS				
			PhD				

Supplemental Online Content: Nonauthor Collaborators
*First name, last name, and suffix (if applicable) are required and will appear in PubMed.

***First Name and Middle Initial(s)**	***Last Name**	***Suffix (eg, Jr, III)**	Academic Degrees	Institution	Location (city, state/province, country)	Role or Contribution, eg, chair, principal investigator	Group (if more than 1 Group listed in the byline) and/or Subgroup (eg, Steering Committee)
			MBBS, PhD,FRACP				
			PhD				
			MBBS, PhD,FRACP				
			GDip Gen Couns				
			PhD				
			PhD				
			MBBS,				
			PhD				
			PhD				
			PhD				
			GDip Gen Couns				
			PhD				
			Grad Dip Clin Res				
			MBBS, PhD				
			MBBS, FRACP				
			PhD				
			PhD				
			GDip Gen Couns				

Supplemental Online Content: Nonauthor Collaborators
*First name, last name, and suffix (if applicable) are required and will appear in PubMed.

***First Name and Middle Initial(s)**	***Last Name**	***Suffix (eg, Jr, III)**	Academic Degrees	Institution	Location (city, state/province, country)	Role or Contribution, eg, chair, principal investigator	Group (if more than 1 Group listed in the byline) and/or Subgroup (eg, Steering Committee)
			MB ChB, MD, FRACP,				
			FACD, FAICD				
			MHSc				
